# Trajectories of mental health outcomes following COVID-19 infection: a prospective longitudinal study

**DOI:** 10.1186/s12889-024-17997-x

**Published:** 2024-02-13

**Authors:** Farzaneh Badinlou, Fatemeh Rahimian, Maria Hedman-Lagerlöf, Tobias Lundgren, Tamar Abzhandadze, Markus Jansson-Fröjmark

**Affiliations:** 1 Centre for Psychiatry Research, Department of Clinical Neuroscience, Karolinska Institute, and Stockholm Health Care Services, Region of Stockholm, Stockholm, Sweden; 2 Medical Unit, Medical Psychology, Women’s Health and Allied Health Professional Theme, Karolinska University Hospital, Stockholm, Sweden; 3 RISE Research Institutes of Sweden, Department of Computer Science, Stockholm, Sweden; 4 Department of Clinical Neuroscience, Institute of Neuroscience and Physiology, Sahlgrenska Academy, University of Gothenburg, Gothenburg, Sweden; 5 Division of Clinical Geriatrics, Department of Neurobiology, Care Sciences and Society, Karolinska Institute, Stockholm, Sweden; 6 Department of Occupational Therapy and Physiotherapy, Sahlgrenska University Hospital, Gothenburg, Sweden

**Keywords:** Anxiety, COVID-19 infection, Depression, Fatigue, Insomnia, Post-COVID impairments

## Abstract

**Background:**

The COVID-19 pandemic has triggered a global mental health crisis. Yet, we know little about the lasting effects of COVID-19 infection on mental health. This prospective longitudinal study aimed to investigate the trajectories of mental health changes in individuals infected with COVID-19 and to identify potential predictors that may influence these changes.

**Methods:**

A web-survey that targeted individuals that had been infected with COVID-19 was used at three time-points: T0 (baseline), T1 (six months), and T2 (twelve months). The survey included demographics, questions related to COVID-19 status, previous psychiatric diagnosis, post-COVID impairments, fatigue, and standardized measures of depression, anxiety, insomnia. Linear mixed models were used to examine changes in depression, anxiety, and insomnia over time and identify factors that impacted trajectories of mental health outcomes.

**Results:**

A total of 236 individuals completed assessments and was included in the longitudinal sample. The participants’ age ranged between 19 and 81 years old (M = 48.71, SD = 10.74). The results revealed notable changes in mental health outcomes over time. The trajectory of depression showed significant improvement over time while the trends in anxiety and insomnia did not exhibit significant changes over time. Younger participants and individuals who experienced severe COVID-19 infection in the acute phase were identified as high-risk groups with worst mental ill-health. The main predictors of the changes in the mental health outcomes were fatigue and post-COVID impairments.

**Conclusions:**

The findings of our study suggest that mental health outcomes following COVID-19 infection exhibit a dynamic pattern over time. The study provides valuable insights into the mental health trajectory following COVID-19 infection, emphasizing the need for ongoing assessment, support, and interventions tailored to the evolving mental health needs of this population.

## Background

The SARS-CoV-2 infection (COVID-19) outbreak has led to mental health problems in the general population [1–3], most profoundly affected by demographical variables such as age, sex, and education as well as pre-exiting mental health problems [4, 5]. In addition, there have been notable changes in mental health problems since the onset of the pandemic, marked by a spike during the first wave of the COVID-19 pandemic and a subsequent decline from the initial baseline assessment to subsequent follow-ups [6–9]. However, levels of mental ill-health have been found to be more elevated in individuals infected with COVID-19 compared to the general population [10], suggesting that the mechanisms through which COVID-19 infection impacts mental health may differ from those observed in the general population.

Studies investigating mental ill-health following COVID-19 infection shed light on a bidirectional association between SARS-CoV-2 infection and mental ill-health [11–15]. However, the impact of COVID-19 infection on mental health becomes more intricate in the context of long-term complaints of COVID-19. Follow-up studies on COVID-19 survivors highlighted the associations between mental ill-health and post-COVID complications [10, 16]. Long term impacts after COVID-19-infection include multi-systemic problems, disabilities, and mental health problems, of which fatigue has emerged as the most reported symptom [17–19]. As many as almost half of all who have a history of probable or confirmed COVID 19-infection experience symptoms after recovery from infection [18], and about 40% of COVID-19 survivors experience fatigue three months after infection, with anxiety, depression and psychiatric comorbidity generating elevated risk [20]. We have previously shown in a cohort study that individuals with a history of probable or confirmed COVID-19 infection/infections are more likely to suffer from mental health problems, with post-COVID impairments and fatigue appearing as the main predictors of mental ill-health [10].

To summarize, available data highlights that COVID 19-patients are a high-risk group for mental ill-health, and points to an interplay between COVID-19-infection and mental ill-health and a possible bi-directional association. However, more knowledge is needed regarding the specific role of post-COVID impairments, especially fatigue, on mental health following COVID-19 infection. Hence, we aimed to investigate the trajectories of mental health changes over time in individuals infected with COVID-19; and to explore potential predictors that may influence these changes.

## Methods

### Participants

In this longitudinal study, we used data from a web-based longitudinal project to study the impacts of COVID-19 infection on a sample of Swedish population [10, 17]. To recruit participants, we used convenience sampling by spreading e-posters on platforms of COVID-19-related Facebook groups, Swedish COVID-organization (Svenska Covidföreningen), and the Karolinska Institutet website. Participants could access the web-survey through an online platform, Research Electronic Data Capture (REDCap), hosted locally at Karolinska institutet [21, 22]. Inclusion criteria were: (*i*) having been infected with COVID-19; (*ii*) age (≥ 18 years); (*iii*) ability to understand Swedish, and use the internet in order to complete the web-survey. The main exclusion criteria in the current study was absence of a prior COVID-19 infection, serving as a key parameter for participating.

The web-based survey was conducted at three time points: (i) at baseline or T0 (February/March 2022), (ii) first follow-up or T1 (September/October 2022), and (iii) second follow-up or T2 (February/March 2023). The number of participants in each cross-sectional data collection varied. A total of 501 participants responded at the baseline (T0), while the response rate was 60.1% at T1 and 57.3% at T2. The longitudinal analysis included 236 (47.1%) participants who completed the survey at all time points.

#### Ethical considerations

The study was approved by the Swedish national ethical board (Dnr 2021–06617-01). Informed consent was obtained from all participants. All procedures utilized in collecting data for the current paper followed the ethical standards of the Helsinki Declaration of 1964 and subsequent amendments [23].

### Measures

#### Time-invariant covariates

Time-invariant covariates in the current study consisted of sociodemographic variables, COVID-19-related variables, and previous psychiatric diagnosis, which were obtained at T0 and assumed to remain unchanged across the study. Sociodemographic variables included age, gender, educational level, work status, and economic status. The ages were grouped by decades.

COVID-19-related variables included time of first infection, hospitalization for COVID-19, being vaccinated against COVID-19, and COVID-19 severity in the acute phase. Time of first infection was measured by a single item in which respondents stated date of first infection (year and month). The variable was dichotomized into during the year 2020 versus during the year 2021 and 2022, in line with our previous study that revealed that individuals who were infected for the first time during the first and second pandemic waves in Sweden (the spring and autumn of 2020) experienced more COVID-19 related problems [17]. Hospitalization for COVID-19 was measured using a single item in which respondents stated on a binary question if they had been hospitalized because of COVID-19 (yes/no). Being vaccinated against COVID-19 was measured with a single item in which respondents indicated if they have received vaccine against COVID-19 on a binary question (yes/no). COVID-19 severity in the acute phase was measured with a 15-item scale describing common symptoms of the COVID-19 infection, namely fever, fatigue, cough, loss of smell and taste, difficulty breathing or shortness of breath, headache/migraine, aches or pain in the body, diarrhoea, skin rash, runny or blocked nose, nausea/vomiting, arrhythmia/palpitations*,* sore throat, cognitive difficulties such as memory and attention, and mental health problems such as sleep problems, depression, and anxiety [24, 25]. Participants rated symptoms that they have had at the beginning of the infection and those the following 4 weeks on a 4-point scale (0 = no, 1 = mild, 2 = moderate, 3 = severe). The respondents’ answers to 15 symptoms of COVID-19 items were summed up to calculate a COVID-19 severity in the acute phase (range 0—45, α = 0.77).

Previous psychiatric diagnosis was assessed using a single item in which respondents stated on a binary question if they had received a psychiatric diagnosis before COVID-19 infection (yes/no).

#### Time-varying covariates

Fatigue and post-COVID impairments were treated as time-varying covariates and assumed to be subject to change across the study. Time-varying covariates were assessed at all three time points (T0, T1, and T2).

##### Fatigue

The Multidimensional Fatigue Inventory (MFI) is a self-report instrument aiming to measure fatigue. The MFI is a 20-item scale and consists of five subscales namely general fatigue, physical fatigue, reduced motivation, reduced activity, and mental fatigue. Each scale contains four items, each rated on 5-point scale, from 1 (Yes, that is true) to 5 (No, that is not true) [26], and total score is calculated by summing all items. Higher scores indicate higher fatigue levels [27], and total score > 60 has been reported as clinically significant fatigue in a previous study [28]. In this study, we used the Swedish version, which has shown adequate psychometric properties [29, 30].

##### Post-covid impairments

Post-covid impairments were measured using a scale consisting of 54 items rated on a 4-point Likert scale (0 = no, 1 = mild, 2 = moderate, 3 = severe), developed and used in our previous studies [10, 17]. Items were categorized into four sub-categories according to the International Classification of Functioning, Disability and Health [31] as impairments in mental functions, impairments in sensory functions and pain, impairments in body system functions, and impairments in activities and participation. The respondents’ answers to each sub-category of post-COVID impairments were summed up and divided by the number of items to obtain the mean for each sub-category.

#### Study outcomes

Mental health variables were considered as study outcomes and consisted of depression, anxiety, and insomnia. Depression was measured with the Patient Health Questionnaire-9 (PHQ-9). The PHQ-9 consists of nine items answered on a four-point Likert scale (0–3), with the total score ranging from 0 to 27 [32–34]. Anxiety was assessed with the General Anxiety Disorder-7 item scale (GAD-7), which contains seven items answered on a four-point Likert scale (0–3) and with a score range from 0 to 21 [35–38]. Insomnia was measured with the Insomnia Severity Index (ISI), that consists of seven items to assess the nature, severity, and impact of insomnia answered on a five-point Likert scale (0–4), the total score ranges from 0 to 28 [39, 40]. The recommended cutoff score of ≥ 10 on each scale was considered as clinically significant depression, anxiety, and insomnia in the current study [33, 36, 40].

### Statistical analysis

Descriptive statistics for sociodemographic variables are provided in terms of percentages, means, and standard deviations for both the baseline and longitudinal samples. Moreover, descriptive statistics for fatigue, post-COVID impairments, and study outcomes are presented in the form of means and standard deviations. Additionally, we computed the intraclass correlation coefficient (ICC) to evaluate variations between the initial baseline and subsequent follow-up assessments for time-varying covariates and study outcomes. An ICC less than 0.4 was categorized as very low, 0.4 to 0.74 as low to acceptable, and 0.75 or higher as excellent [41].

To assess the potential impact of the covariates, we used mixed-effects models, which are well-suited statistical tools for longitudinal data analysis. Participants were included in the model only if data from all three measurements were available for a given mental health outcome. The alpha value of the two-tailed level of significance was set at 0.05.

We ran linear mixed models with random intercepts to examine differences in mental health outcomes (PHQ-9, GAD-7, and ISI scores) over time with adjustment for sociodemographic variables, COVID-19-related variables, and previous psychiatric diagnosis. Furthermore, we ran linear mixed models to identify factors that impacted the trajectories of depression, anxiety, and insomnia by including both time-invariant and time-varying covariates in the model. We considered AIC (Akaike Information Criterion) and BIC (Bayesian Information Criterion) as model fit in the current study. A lower AIC or BIC value indicates a better fit. Statistical analysis was performed using *statsmodel* library (version 0.13.5) in Python, and IBM Statistical Software Package of Social Science (SPSS; version 26).

## Results

### Descriptive statistics

Descriptive statistics for sociodemographic variables are presented for the baseline sample and the longitudinal sample (Table 1). We compared whether sociodemographic variables could predict whether participants completed surveys at each time point. The results showed that there were no significant differences between participants who completed the survey at all time points and those who did not complete the survey regarding sex, age, education level, marital status, work status, and economic status.

**Table 1:** Sociodemographic characteristics of the baseline sample and the longitudinal sample

	Baseline sample (*n* = 501)		Longitudinal sample (*n* = 236)	
Sociodemographic variables	*N*	%	*N*	%
Age, years, mean (± SD)	47.67 (10.57)		48.71 (10.74)	
Gender				
Female	441	88	210	89
Education				
Pre-secondary/Secondary	152	30.3	65	27.5
University/Post-graduate	349	69.7	171	72.5
Marital status				
Single	104	20.8	47	19.9
Married	209	41.7	103	43.6
In a relationship	144	28.7	66	28
Divorced/separated	40	8	18	7.6
Widowed	4	.8	2	.9
Work status				
Working full time/part time	330	65.9	154	65.3
Unemployed/unpaid work	15	3	7	3
Retired	23	4.6	13	5.5
Parental leave	5	1	0	0
Sick leave	107	21.4	56	23.7
Student	21	4.2	6	2.6
Self-rated economic status				
Below average	84	16.8	34	14.4
Average	243	48.5	114	48.3
Above average	174	34.7	88	37.3

The majority of the longitudinal sample had been infected with COVID-19 for the first time during the year 2020 (69.5%), had not been hospitalized for COVID-19 (85%), and had been vaccinated against COVID-19 (83.9%). The average severity of COVID-19 in the acute phase was 24.7 (standard deviation = 7.8, ranging from 4 to 44). Furthermore, 27.6% of the respondents reported that they had received a psychiatric diagnosis before COVID-19 infection.

**Fig. 1:**
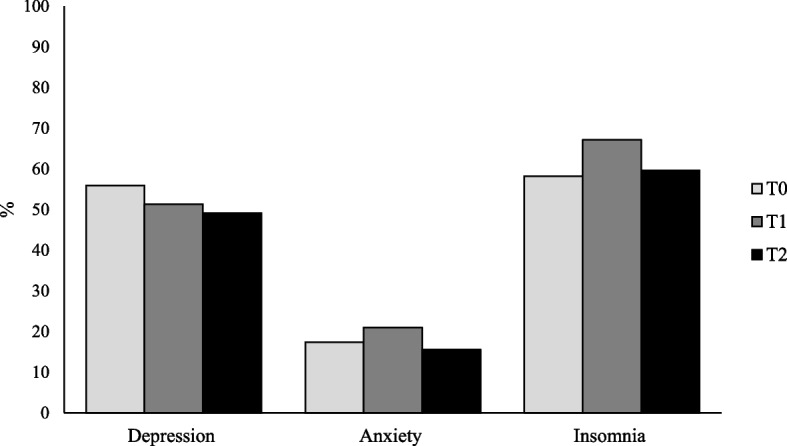
Proportion of people reporting clinically significant levels of depression, anxiety, and insomnia over time

Table 2 presents descriptive statistics for fatigue, post-COVID impairments, and mental health outcomes over time in the longitudinal sample. A decline in mean total fatigue score was observed from T0 to T2. In addition, the prevalence of fatigue (scores > 60 points) decreased constantly from 90.5% to 83.5% from T0 to T2. The mean values of post-COVID impairments decreased slightly from T0 to T2. Figure 1 presents the proportion of clinically significant levels of depression (≥ 10 points on PHQ-9), anxiety (≥ 10 points on GAD-7), and insomnia (≥ 10 points on ISI) over time.

**Table 2:** Descriptive statistics for fatigue, post-COVID impairments, and mental health outcomes over the three measurement points (*N* = 236)

		T0 Baseline	T1 First follow up	T2 Second follow up	ICC
	Range	Mean (SD)	Mean (SD)	Mean (SD)	
MFI-20 (Total score)	20–100	77.87 (12.77)	75.11 (15. 31)	74.77 (16.26)	.69
Post-COVID impairments					
Impairments in mental functions	0–3	1.33 (.66)	1.23 (.66)	1.25 (.67)	.71
Impairments in sensory functions and pain	0–3	1.14 (.63)	1 (.67)	1.02 (.68)	.78
Impairments in body system functions	0–3	1.05 (.55)	.92 (.58)	.94(.59)	.80
Impairments in activities and participation	0–3	1.42 (.75)	1.27 (.82)	1.34 (.84)	.51
Mental health outcomes					
PHQ-9	0–27	10.84 (5.46)	10.4 (5.54)	9.91 (5.52)	.67
GAD-7	0–21	5.62 (4.32)	6.11 (4.48)	5.47 (4.19)	.66
ISI	0–28	11.68 (7)	12.94 (6.94)	11.77 (6.67)	.68

*ICC* Intraclass Correlation Coefficient, *SD* standard deviation, *MFI-20* Multidimensional Fatigue Inventory-20, *PHQ-9* Patient health questionnaire-9, *GAD-7* Generalised Anxiety Disorder-7 item scale, *ISI* Insomnia Severity Index

### Predictors of the trajectories of depression, anxiety, and insomnia

Adjusted estimates of the changes in depression, anxiety, and insomnia scores over time from the linear mixed models are shown in Table 3. The results demonstrated a significant decline in depression over time, while no significant changes were observed in anxiety and insomnia. We also studied the interaction between time and other variables including sociodemographic variables, COVID-19-related variables, and previous psychiatric diagnosis, but none of the interactions proved significant. The model fit metrics (AIC and BIC) suggested that adding the interactions only diminished the model fit.

**Table 3:** Adjusted estimates of the change in depression, anxiety, and insomnia over time from linear mixed models

	Adjusted^a^		Adjusted^b^		Adjusted^c^	
	Coef	95%CI	Coef	95%CI	Coef	95%CI
Depression (PHQ-9)						
Time	-.003*	[-.006, -.001]	-.003***	[-.005, -.001]	-.003***	[-.005, -.002]
Anxiety (GAD-7)						
Time	-.002	[-.005, .001]	-.001	[-.003, .001]	-.001	[-.003, .001]
Insomnia (ISI)						
Time	.000	[-.003,.003]	.000	[-.002, .002]	.000	[-.002, .002]

*PHQ-9* Patient health questionnaire-9, *GAD-7* Generalised Anxiety Disorder-7 item scale, *ISI* Insomnia Severity Index, *Coef* regression coefficient, *CI* Confidence interval

^a^Linear mixed model with random intercepts adjusted for sociodemographic variables

^b^Linear mixed models with random intercepts adjusted for sociodemographic and COVID-19-related variables

^c^Linear mixed models with random intercepts adjusted for sociodemographic variables, COVID-19-related variables, and previous psychiatric diagnosis

^*^ *p* < .05. **p < .01. ***p < .001

Table 4 presents estimates derived from the linear mixed models examining the associations between sociodemographic variables, COVID-19-related variables, previous psychiatric diagnosis and the outcome variables. Separate models were employed for depression, anxiety, and insomnia. The findings indicated that younger adults and individuals experiencing more severe COVID-19 infection in the acute phase exhibited poorer mental health outcomes (Table 4).

**Table 4:** Adjusted estimates from individual multivariable linear mixed models predicting depression, anxiety, and insomnia

	**Depression (PHQ-9)**		**Anxiety (GAD-7)**		**Insomnia (ISI)**	
	Coef	95%CI	Coef	95%CI	Coef	95%CI
Age group	-.016**	[-.028, -.005]	-.035***	[-.048, -.022]	.017*	[.003,.031]
Gender	-.009	[-.048, .030]	-.007	[-.049, .036]	.019	[-.027, .065]
Education	-.022	[-.050, .006]	-.004	[-.035, .026]	-.035*	[-.068, -.003]
Marital status	-.011	[-.040, .018]	.002	[-.030, .033]	-.002	[-.036, .032]
Work status	-.043**	[-.071, -.015]	-.014	[-.045, .016]	.002	[-.032, .035]
Economic status	-.020	[-.048, .008]	-.021	[-.052, .009]	-.008	[-.041, .024]
Being vaccinated against COVID-19	-.027	[-.061, .007]	-.035	[-.072, .002]	-.034	[-.074, .006]
Hospitalization for COVID-19	.013	[-.025, .051]	.009	[-.032, .050]	.056*	[.012, .101]
Time of first infection	.014	[-.012, .040]	-.028	[-.057, .000]	.003	[-.028, .033]
Severity of COVID-19 infection	.356***	[.281, .431]	.276***	[.195, .356]	.360***	[.272, .447]
Pevious psychiatry diagnosis	.017	[-.015, .049]	.022	[-.012, .057]	.025	[-.013, .062]
Model fit indices						
AIC	-.319		-.163		-.003	
BIC	-242.030		-93.004		60.618	

*PHQ-9* Patient Health Questionnaire-9, *GAD-7* Generalised Anxiety Disorder-7 item scale, *ISI* Insomnia Severity Index, *Coef* regression coefficient, *CI* Confidence interval, *AIC* Akaike Information Criterion, *BIC* Bayesian Information Criterion

^*^ *p* < .05. **p < .01. ***p < .001

The outcome of the linear mixed model, examining the associations between fatigue, post-COVID impairments and the outcome variables (depression, anxiety, and insomnia) are presented in Table 5. We conducted the analysis at the individual level, ensuring implicit adjustment for sociodemographic factors, COVID-related variables, and previous psychiatric diagnosis. The results showed that fatigue appeared to be a significant predictor for all outcomes, and impairments in mental function were an additional significant predictor for depression and anxiety. Both variables had a positive impact on all outcomes, with fatigue being the strongest predictor.

**Table 5:** Adjusted estimates from individual multivariable linear mixed models of associations between fatigue and post-COVID impairments and depression, anxiety, and insomnia

	**Depression (PHQ-9)**		**Anxiety (GAD-7)**		**Insomnia (ISI)**	
	Coef	95%CI	Coef	95%CI	Coef	95%CI
Fixed effects						
Intercept	-.049	[-.105, .007]	.160	[-.096, .034]	.129**	[.050, .208]
Impairments in mental functions	.201***	[.118, .284]	-.019**	[.064, .256]	.106	[-.008, .219]
Impairments in sensory functions and pain	.053	[-.051, .156]	.021	[-.139, .101]	.091	[-.052, .235]
Impairments in body system functions	.008	[-.123, .139]	.016	[-.131, .172]	.066	[-.116, .249]
Impairments in activities and participation	.069	[-.005, .143]	.319	[-.070, .103]	.032	[-.070, .135]
Fatigue	.429***	[.344, .514]	.160***	.223, .415]	.255***	[.140, .371]
Random effects						
Individual intercept	.011		.022		.031	
Model fit indices						
AIC	-1.134		-.848		-.488	
BIC	-678.014		-505.475		-277.649	

Patient Health Questionnaire-9, *GAD-7* Generalised Anxiety Disorder 7-item scale, *ISI* Insomnia Severity Index, *Coef* coefficient, *CI* Confidence interval, *AIC* Akaike Information Criterion, *BIC* Bayesian Information Criterion

^*^ *p* < .05. **p < .01. ***p < .001

## Discussion

We investigated trajectories of mental health outcomes over one year in Swedish adults with COVID-19, using a three-wave survey. Our results demonstrated a significant decline in depression over time, while small, nonsignificant fluctuations were observed in anxiety and insomnia. Furthermore, younger adults and individuals who experienced more severe COVID-19 infections in the acute phase at baseline exhibited poorer mental health outcomes. Fatigue emerged as the most consistent predictor of changes in depression, anxiety, and insomnia. Impairments in mental function due to COVID-19 infection appeared as one of the main predictors of changes in depression and anxiety but not insomnia.

In this study, levels of depression decreased constantly, anxiety exhibited a slight increase, followed by a subsequent decrease, remaining below the baseline level, and insomnia increased slightly and then decreased, consistently remaining above the baseline level. Our findings are in line with previous studies indicating that mental health problems remained more prevalent among individuals who have had COVID-19 infection [42–47]. However, symptoms of depression and anxiety decreased over time regardless of the initial severity of the disease [48, 49]. There are several possible explanations for these findings. Firstly, depression and anxiety symptoms have shown a decreasing trend in the general population, including our participants, during the COVID-19 pandemic [50]. During the COVID-19 pandemic in Sweden, individuals were encouraged to work from home when possible. Additionally, gatherings of more than 50 people were prohibited, many businesses and higher education institutions voluntarily transitioned to video conferencing, and non-essential travel was significantly reduced. However, at the onset of the study period in February 2022, Swedish authorities changed their strategies in response to the pandemic similar to other European nations, leading to the lifting of the majority of COVID-19 restrictions [51]. The relaxation or removal of COVID-19-related restrictions, facilitated by the global vaccination campaign, has enabled people to resume their pre-pandemic lifestyles and activities. This transition may have alleviated depression and anxiety symptoms, as individuals restore a sense of normality and participate in activities that provide them with joy and fulfillment. Another potential factor is the enactment of mental health recovery strategies by policymakers in various countries, including Sweden. Strategies include initiatives to monitor, inform, educate, intervene, and research mental health issues in society [52], and efforts target both immediate and long-term mental health outcomes. The third possible explanation for these findings is sustained recovery of COVID-19-related persistent symptoms over time. A substantial proportion of individuals infected with COVID-19 reports experiencing at least one moderate-to-severe impairment due to COVID-19 infection, with fatigue being the most commonly reported symptom [17, 53–61]. Furthermore, our previous cross-sectional study revealed that post-COVID impairments and fatigue emerged as significant predictors of mental ill-health in individuals who were infected with COVID-19 infection [10]. However, a progressive improvement has been observed in a wide array of symptoms over time [48, 62, 63]. Our study results indicate that impairments in mental function and fatigue affect depression and anxiety changes over time. These factors shape the dynamics of depression and anxiety and are key for their longitudinal course, thus, managing these complaints may improve mental well-being. In summary, the reduction of symptoms of depression and anxiety observed in this study may be linked to the global recovery from the COVID-19 pandemic and the improvement of post-COVID complaints, especially fatigue.

We found that insomnia, unlike depression and anxiety, increased slightly before decreasing during COVID-19 recovery, but remained above baseline throughout the study period. This indicates a complex interaction of factors affecting sleep quality in this population. These findings are consistent with a previous study which demonstrated a decrease in the symptoms of depression and anxiety whereas increased symptoms of insomnia among COVID-19 patients over time [64]. Additionally, another study indicated that there was no significant change in insomnia over time among COVID-19 patients [65]. Several factors may contribute to this pattern of insomnia exhibiting a different pattern than other symptoms of mental ill-health. First, the rates of insomnia increased significantly during the COVID-19 pandemic like other mental health issues [4] and the prevalence of insomnia was higher in COVID-19 infected patients compared with the general population [10, 64, 66]. The initial increase in insomnia could be attributed to the physiological and psychological effects of the acute phase of COVID-19 infection and the side effects of COVID-19-related medications which disrupted sleep quality and quantity during the early stages of recovery and increased the risk of developing chronic insomnia later after recovery [55, 67–69]. Second, sleep-related problems were reported as one of the most common remaining symptoms experienced after recovering from COVID-19 [17, 48]. However, post-COVID impairments did not significantly contribute to changes in insomnia following COVID-19 infection in the current study. Interestingly, it was observed that fatigue emerged as a significant predictor in relation to insomnia. The co-occurrence of fatigue and insomnia has previously been found to be highly prevalent among individuals following recovery from COVID-19 infection [70]. This finding suggests that these two symptoms frequently manifest together in individuals who have experienced the illness. Additionally, several studies have highlighted the presence of fluctuations and relapses in post-COVID-19 fatigue over time [48, 71]. The interplay between fatigue and insomnia can create a vicious cycle, particularly among patients with long-term COVID [72]. Fatigue can contribute to increased sleep difficulties, while insomnia can exacerbate feelings of fatigue and prolong recovery. This bidirectional relationship between fatigue and insomnia may lead to a chronic cycle of symptoms and further impact overall well-being. Lastly, it is essential to consider the bidirectional relationship between mental health and sleep. Insomnia can exacerbate persistent symptoms of depression and anxiety, while these mental health conditions can also contribute to sleep disturbances [73]. To reduce symptoms of depression and anxiety may help to improve insomnia in COVID-19 survivors, and better mental health and coping skills can improve sleep quality. Insomnia needs ongoing assessment and treatment in individuals infected with COVID-19. In addition, addressing fatigue and mood may also reduce insomnia.

Further analysis revealed that younger adults and individuals who experienced more severe COVID-19 infections in the acute phase exhibited poorer mental health outcomes. Previous studies have demonstrated that younger adults have been more profoundly affected by the pandemic and exhibit higher levels of mental health problems [4, 74, 75]. Younger adults, despite primarily experiencing mild COVID-19 infections, faced greater challenges related to the long-term impacts of COVID-19 infection, which significantly disrupted their presentation and performance in their work, education, and daily activities. Hence, it can be concluded that young adults remain within the at-risk group for mental ill-health following COVID-19 infection. Moreover, prior research has consistently demonstrated that the severity of COVID-19 infection in the acute phase is strongly linked to persistent post-infection symptoms [53, 55, 56, 61], emerging as the most robust predictor of post-COVID impairments (Badinlou et al., 2023). Additionally, it has been shown to contribute to higher levels of mental ill-health following COVID-19 infection [10]. Therefore, it is reasonable to conclude that individuals who experienced severe COVID-19 infection in the acute phase continue to be at risk for mental ill-health. These findings highlight the importance of considering sociodemographic and COVID-19-related factors when examining the impact of COVID-19 on mental well-being.

The primary objective of our study was to explore the potential trajectories of mental health changes following COVID-19 infection. To achieve this goal, we focused on minimizing the risk of overlooking real effects (i.e., Type II errors) rather than strictly controlling the risk of falsely identifying effects (i.e., Type I errors). This approach was deemed more appropriate for our exploratory research, since it allowed us to prioritize detecting patterns in the data, even if it meant there was a slightly increased risk of overlooking some effects.

The current study has several practical implications. First, understanding changes in mental health outcomes following COVID-19 infection and identifying risk factors could help healthcare providers to develop targeted interventions to support those who have been infected and may be experiencing psychological problems. Second, the findings provide policymakers with evidence-based insights to implement strategies that can mitigate the long-term mental health impact of the COVID-19 infection, and promote mental well-being in individuals infected with COVID-19, even those who experienced a mild infection. Finally, it contributes to the broader body of research on the mental health consequences of infectious diseases, potentially guiding future pandemic preparedness and response efforts.

Nevertheless, it is important to interpret the results of the study in the context of its limitations and consider potentially confounding factors. First, the current study uses self-reported data for mental health outcomes, which may be biased or inaccurate compared to clinical assessments. Second, it uses a convenience sample, which may limit the generalizability of the findings. Hence, future studies should use more representative samples. Third, it may suffer from non-response bias, as participants who continued or dropped out may differ in important ways. Fourth, we could not establish causality between COVID-19 infection and mental health changes, as there may be other confounding factors. Fifth, it lacks a control group that did not contract COVID-19, which makes it hard to isolate the effects of the infection on mental health. Sixth, majority of participant in the current study was female, introducing the possibility of gender-related biases and potentially limiting the generalizability of the findings to a more balanced demographic.

## Conclusions

This study provides a longitudinal perspective on mental health issues following COVID-19 infection, shedding light on the dynamic nature of mental health outcomes over time and underscoring the importance of continued support and interventions tailored to the changing mental health requirements of this affected population. Further research is needed to understand the underlying factors contributing to these changes and to develop targeted interventions for individuals experiencing persistent mental health symptoms.

## Data Availability

The datasets used and/or analyzed during the current study are available from the corresponding author on reasonable request.
